# Self-organized integration vs. self-organized disintegration: an unfinished study 

**DOI:** 10.3389/fnetp.2025.1662127

**Published:** 2025-10-20

**Authors:** Juval Portugali

**Affiliations:** Department of Geography and the Human Environment, Tel Aviv University, Tel Aviv, Israel

**Keywords:** network physiology, synergetics, cities, complexity, artifacts, memory

## Abstract

This paper refers to an issue Haken and myself were discussing, started to work on, prepared a preliminary draft, but never managed to complete and transform it into a full-scale study and publication. Here, in memoriam of Hermann Haken, my dear friend and colleague for many years, I present it as it is – an unfinished study with some innovative ideas that will have to be further elaborated in the future.

## 1 Introduction

Network physiology (NP) is a new field of research (Ivanov 2021) whose main focus is the “human organism” and the fact that it “comprises various physiological and organ systems, each with its own structural organization and functional complexity, leading to complex, transient, fluctuating and nonlinear output dynamics”, namely, to a complex system. The main challenge of NP is to explore the “coordinated network interactions among systems and sub-systems” in order to avoid the “dysfunction of individual systems” and “cascade of failures leading to a breakdown and collapse of the entire organism”. Such collapse processes typify also other complex systems that similarly to the human organism are comprised of several subsystems each of which is itself a complex system. Cities and systems of cities are a case in point.

The aim of this paper is to shed light on the dynamics of this process of breakdown and collapse of the entire organism/system from the perspective of *complexity theories of cities* (CTC) – A domain of research that applied the various theories of complexity to the study of cities, their planning and design (Portugali, 2000; 2011; Haken and Portugali, 2021). More specifically, as shown below, the study of cities as complex systems exposed, firstly, a general lacuna in complexity theory at large concerning the dynamics of collapses. Secondly, that in the case of cities there is order in this process of collapse, similar in several ways to the order that emerges in complex systems by means of self-organization. Thirdly, that this ordered collapse process typify other complex systems, including NP of human organisms.

The discussion below thus evolves as follows: It starts (Sect. 1) by introducing the above noted lacuna, the essence of which is a distinction between two forms of self-organization: self-organized integration (SOI) and self-organized disintegration (SOD). Next, it introduces two past studies on system collapse (Sect. 2). Sect. Three that follows, presents our synergetics perspective on SOI vs SOD, first in relations to the role of the control parameter and second in relations to the two foundations of synergetics: the microscopic bottom-up and the macroscopic top-down. Sect. Four describe two case studies that provided inspiration to the notion of SOI vs SOD. Sect. Five suggests a distinction between two kinds of complex systems – with and without memory and then shows that processes of SOD are typical complex systems with memory. As indicated by the title of this paper and its Prolegomenon, this is an unfinished study. Sect. Six is an epilogue suggesting possible research directions to complete this study. The paper concludes by exploring the implications to the domain of networks physiology and by posing directions for further research.

## 2 The lacuna

**FIGURE 1:**
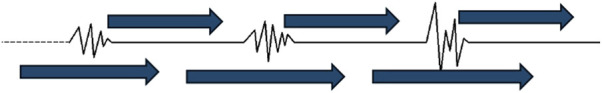
Top: The evolution of complex systems, urban systems included, is characterized by long periods of steady state interrupted by short events of sharp fluctuations and chaos, out of which the system emerges to a new steady state and so on. As indicated by the arrows, the focus of interest was traditionally on the processes from disorder to order. *Bottom*: An alternative way of looking at the processes: from ordered steady state to disorder characterized by strong fluctuations and then to ordered steady state again.

**FIGURE 2:**
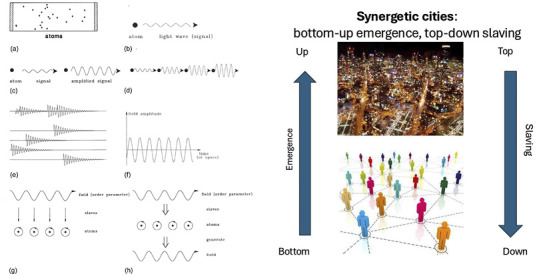
Left. The laser paradigm. **(a)** A typical setup of a gas laser. A glass tube is filled with gas atoms and two mirrors are mounted at its end faces. The gas atoms are excited by an electric discharge. Through one of the semi-reflecting mirrors, the laser light is emitted. **(b)** An excited atom emits light wave (signal). **(c)** When the light wave hits an excited atom it may cause the atom to amplify the original light wave. **(d)** A cascade of amplifying processes. **(e)** The incoherent superposition of amplified light waves produces still rather irregular light emission (as in a conventional lamp). When sufficiently many waves are amplified they strongly compete for further energetic supply. That wave that amplifies fastest wins the competition initiating laser action. **(f)** In the laser, the field amplitude is represented by a sinusoidal wave with practically stable amplitude and only small phase fluctuations. The result: a highly ordered, i.e., coherent, light wave is generated. **(g)** Illustration of the slaving principle. The field acts as an order parameter and prescribes the motion of the electrons in the atoms. The motion of the electrons is thus “enslaved” by the field. **(h)** Illustration of circular causality. On the one hand, the field acting as order parameter enslaves the atoms. On the other hand, the atoms by their stimulated emission generate the field. Right. Synergetic Cities: in analogy to the laser paradigm, the interaction between the urban agents gives rise to an urban OP that … and so on in circular causality. Source: Haken and Portugali (2021).

Complex systems are typified by long periods of steady state (StS) interrupted by short periods of strong fluctuations and chaos that often lead to a new StS and so on (Figure 1). According to Haken’s synergetics, the long periods of StS are a consequence of a process by which the interaction between the parts of the system gives rise to an order parameter (OP) that enslaves the parts and thus keeps the systems in steady state. The common way to study this dynamic is to focus on the way the interaction between the parts of the system during its period of strong fluctuations and chaos leads to the emergence of order and a steady state. This approach is at the core of the four paradigms of synergetics described below in Sect. 3.2: The laser, pattern formation, pattern recognition and finger movement paradigms. Of the latter, the synergetics laser paradigm, illustrated in Figure 2 *left*, is probably the most typical illustration of this dynamics. As can be seen in Figure 2 *left*, the focus of interest here is on the emergence of order, that is to say, on the steady state.

This is in fact common to the majority (all) of research in this field of complexity that focuses on the process of integration, where autonomous elements that exist outside each other enter an interactive dynamics that turns them into a single integrated system. This is the essence of Haken’s Synergetics as noted, of the dissipative structures of Prigogine, and of Mandelbrot’s fractal theory. The titles of the books *Order out of Chaos* by Prigogine and Stengers (1984) and of Stuart Kauffman (1993), Kauffman (1995) *The Origins of Order* also suggest the emergence of order from chaos. This holds true for the concept of self-organization, for all models of cellular automata and agent-based models, and as we have seen above and will see further, in the discussion of network dynamics: the main effort is to explain the transition from local to global networks, from disorder to order.

This holds true also to the domain of CTC that emerged following the application of the various theories of complexity to the study of cities (Portugali, 2000; 2011). While each of these applications emphasizes a different aspect of the dynamics of cities, they all share the common view that “cities emerge from the bottom up” – a view and a statement that became the mantra of this domain. For example, in the case of *Synergetic Cities* (Haken and Portugali, 2021), in analogy to the laser paradigm, the interaction between the urban agents – the parts of the urban system – gives rise to an urban order parameter that “enslaves” (describes and prescribes) the behavior and action of the urban agents and so on in circular causality (Figure 2, Right).

Interestingly, this emphasize on bottom-up characterizes also Weidlich’s (1999) attempt to study complex systems that comprise various sub-systems, each with its own structural organization and functional complexity, of which cities and systems of cities form typical examples. Thus he writes (Weidlich, 1999):

If in a system of nonlinear equations of motion for many variables these variables can be separated into slow ones and fast ones, a few of the slow variables. . . are predestined to become “order parameters” dominating the dynamics of the whole system on the macroscale.

Applied to urban systems, the fast variables typify the local urban microlevel small independent cities, whereas the slow processes typify the macrolevel metropolises and regional systems of cities. The relations between the slow and the fast processes are in line with the slaving principle: on the one hand, the regional system “serves as the environment and the boundary condition under which each local urban microstructure evolves. On the other hand, the regional macrostructure is the global resultant of [the interaction between] many local structures” (Weidlich ibid). The common view is thus to follow the self-organized process by which the interaction between the un-related parts of the system (i.e., a state of chaos) integrates into a systemic whole; in short, to follow the process of *self-organized integration* (SOI).

But there is a lacuna in this view on cities and by implication in the study of complex systems at large: there is another way to look at the above rhythm, as in Figure 1 Bottom: Namely, to start from the steady state period and to follow the process of disintegration. When this was done it was found that there is order in this process: namely, similarly to SOI the process evolves by means of self-organization as self-organized disintegration (SOD). To be sure, there have been attempts to follow the process of systemic disintegration and collapse and two such attempts are described next. However, as also shown next, they go half way only – they describe a systemic collapse but not the process of SOD associated with it. So the lacuna is still there.

## 3 On system collapse

As just noted, while the main focus was on SOI, there were a few studies who did delve into the “reverse” process that leads to fragmentation, that is, the transition from order to disorder and chaos. In what follows I present two such sturdies – the first, from the past, by member of the so called *Club of Rome* in connection with environmental problems, while in recent years, by Shlomo Havlin and his group and their notion of the ‘cascading effect’.

### 3.1 The club of Rome

**FIGURE 3:**
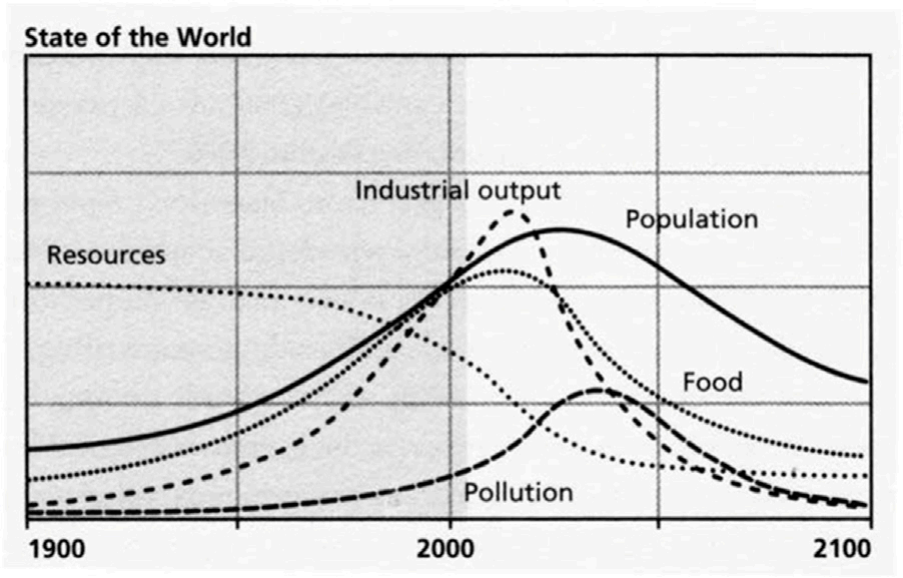
Collapse of systems as a result of the relationship between population growth, industrial production, raw materials, pollution and food: population growth and industrial growth lead to overexploitation of resources and environmental pollution, which result in systems’ collapse. Source: Meadows et al., 1972.

The key question of the Club of Rome members (Meadows et al., 1972) was reversed to the key question of students of complex systems: what is the dynamics of the breakdown of a system? At the center of their discussion were the system’s variables, such as demographic growth rates, economic growth rates, rates of exploitation of natural resources, and the like. These variables, as can be understood, result from the behavior of the parts of the system—the behavior of individuals in society, the behavior of public bodies, and more. According to Synergetics, when the system is in a steady state, these variables are moving in a certain rhythm controlled by the order parameter of the system, ensuring that its dynamics remain within the range of its carrying capacity. According to the Club of Rome members, the problem begins when one of the system variables, such as the demographic growth rate, goes beyond the carrying capacity. The result is system collapse (Figure 3).

### 3.2 The cascading effect

**FIGURE 4:**
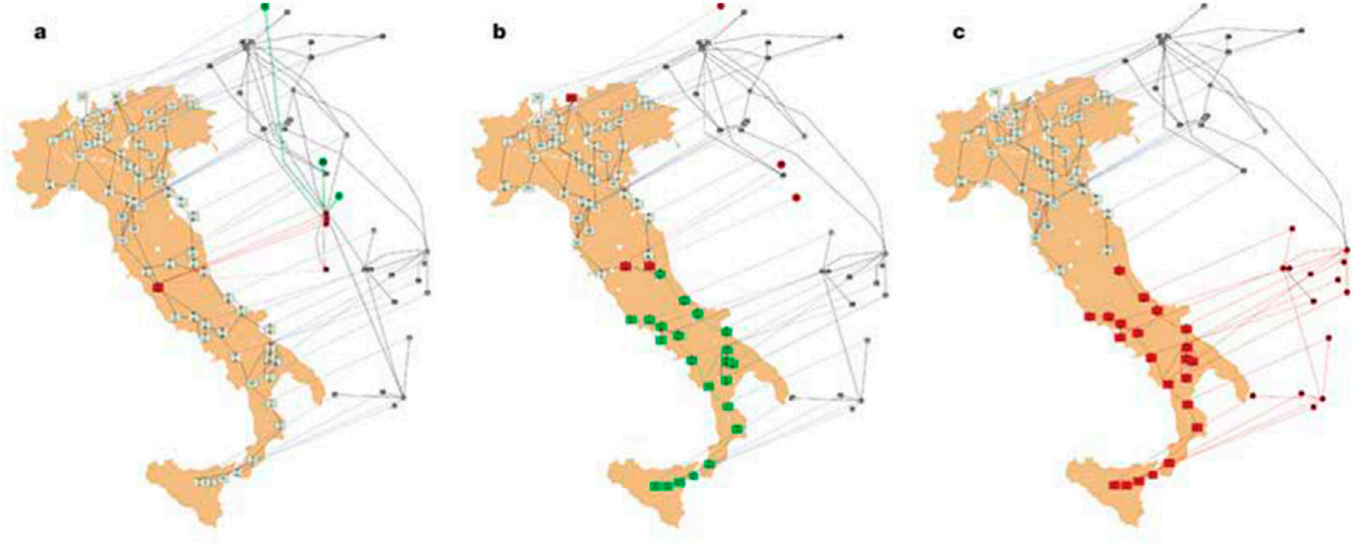
One power station in the electricity network collapses (**a**-left), and as a result several hubs in the Internet network went out of action. Additional power plants whose operation relies on the Internet are also collapsing (**b**-center) and knocking down additional internet hubs, the lack of which leads to the collapse of the power plants that rely on them (**c**-right), and so on. Source: Buldyrev et al. (2010).

In recent years, the issue of system collapse was studied by Havlin and his research group (Buldyrev et al., 2010), focusing mainly on the effects of systems’ interdependence. In their approach, there are two innovations: firstly, they demonstrate that many complex systems are characterized, among other things, by the proliferation of networks and interdependencies between them. Secondly, as a result of this, when one network fails (for example, the power grid), it often triggers the failure of other networks (such as transportation, etc.) that depend on it. The term used to describe this process is the *cascading effect*. A case in point was the ‘blackout’ that occurred in Italy in September 2003: the failure of one power station leads to the cascading failure of both the internet and electricity networks due to the existing interdependencies between the two networks (Figure 4).

It is important to note that while Buldyrev et al. study (ibid) refers to the specific case study of the September 2003 ‘blackout’ in Italy, at its core is a general approach relevant to several domains including the domain of NP: As noted by Ivanov and Bartsch (2014), Buldyrev et al. study applies to the Italian case-study the general approach of statistical physics of networks that refers to ‘networks with complex topologies’ focusing ‘on critical transitions due to failure in the coupling of interdependent networks – an issue which is central also to NP.

But there still is a lacuna in these studies too as the detailed dynamics and outcome of the disintegration process is not addressed. In both the collapse is an abrupt event, not a process. In the case of the Club of Rome, it is due to a situation by which one of the system variables goes beyond the environmental carrying capacity; in the case of Italy, it is due to the breakdown of the electricity subsystem, with the implication that once the electricity network is being restored, the system will return to is previous steady state.

## 4 A synergetics perspective on SOI vs SOD

### 4.1 The two facets of the control parameter

An alternative approach to studying the processes of integration and disintegration in complex self-organizational systems was suggested by Portugali & Haken (unpublished). Our starting point was a network that typically consists of nodes and links between them. The number of links in such a network is a variable (parameter) that can grow, decrease, or remain constant. This variable is called the ‘control parameter’ in the language of Synergetics. For example, in a network that undergoes self-replication, the control parameter is in a growth process. In fact, the discussion of self-organization in complex networks has mostly focused on the situation where the control parameter is in a growth process. However, this is a specific case, as the control parameter may not only grow but also decrease.

In our model, the control parameter is in a process of growth or decay. The important point is that when the change in the control parameter crosses a certain threshold, whether in the growth or decay process, a phase transition occurs in the network’s structure. This is known as a beyond-the-critical-point phase transition: Suddenly, the system’s structure changes dramatically. So far, network research has mainly focused on the growth process of the control parameter. This is so in Erdős and Rényi, (1959) random graph models, as well as in Watts' small-world model (1999) and later in Barabási’s (2002) model: when the control parameter crosses a certain threshold, the system transitions from a local network to a small-world or from a local network to a global network.

A diametrically symmetric process, occurs when the control parameter is in a decay process. Imagine a global urban network characterized by a hierarchical structure of different-sized settlements. If we start a random process in which, in each iteration, one or more links in the system are being disconnected, we can observe that at the beginning of the process, the influence is quantitative, meaning the number of links in the system indeed decreases, but the system’s structure remains stable. Then, suddenly, a further and minor decrease in the number of links leads to a qualitative change – a dramatic change in the network’s structure.

How does the system disintegrate, and how does it self-organizes itself in the process? The various complexity theories do not address this question, including the above noted theory of Havlin’s cascading effect. They conclude with the collapse of the system but do not explore the associated processes of disintegration-selforganization. The cascading effect, in its beyond-the-critical-point phase transition, does describe the dynamics of the catastrophe, but it does not continue to address its possible dynamics.

The answer suggested here is that it depends on the kind of the complex system concerned, or more specifically, on the realization that there are different kinds of complex systems, each reacting differently to the decrease of the system’s control parameter and thus disintegrates differently. The first to refer to different kinds of complex systems was Hermann Haken in two books: *Synergetic computers and cognition: A top-down approach to neural nets* (Haken 1991/2004) and *Information and self-organization: A macroscopic approach to complex systems* (Haken, 1988). In the latter book he made a distinction between Synergetics’ first and second foundations.

A second realization that there are different kinds of complex systems was the notion of *complex adaptive systems* (CAS) introduced by Gell-Mann (1994) and Holland (1995), implying a distinction between material complex system and living complex systems which are adaptive. While their notion of CAS, became a dominant paradigm of complex systems, Haken’s first and second foundations remained confined to Synergetics; yet, they are essential to understand the distinction between the bottom-up SOI vs. he top-down SOD.

### 4.2 The first and second foundations of synergetics

**FIGURE 5:**
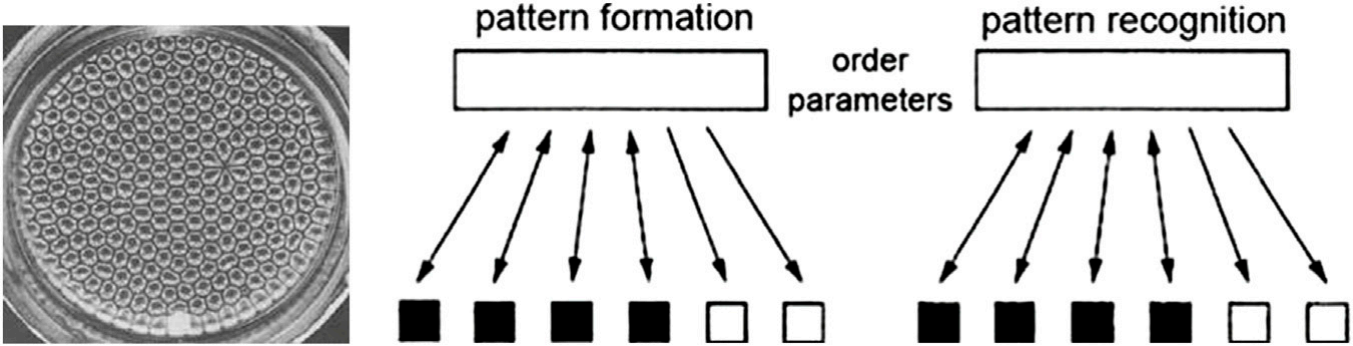
Left: Bénard convection cells as an example of pattern formation – a macroscopic pattern of hexagons emerges by means of self-organization. Right: Haken’s analogy between pattern formation and pattern recognition. In both processes, a configuration of some parts of a system gives rise to an order parameter which enslaves the rest of the parts and brings the whole system to an ordered state. Source: Haken (1991).

**FIGURE 6:**
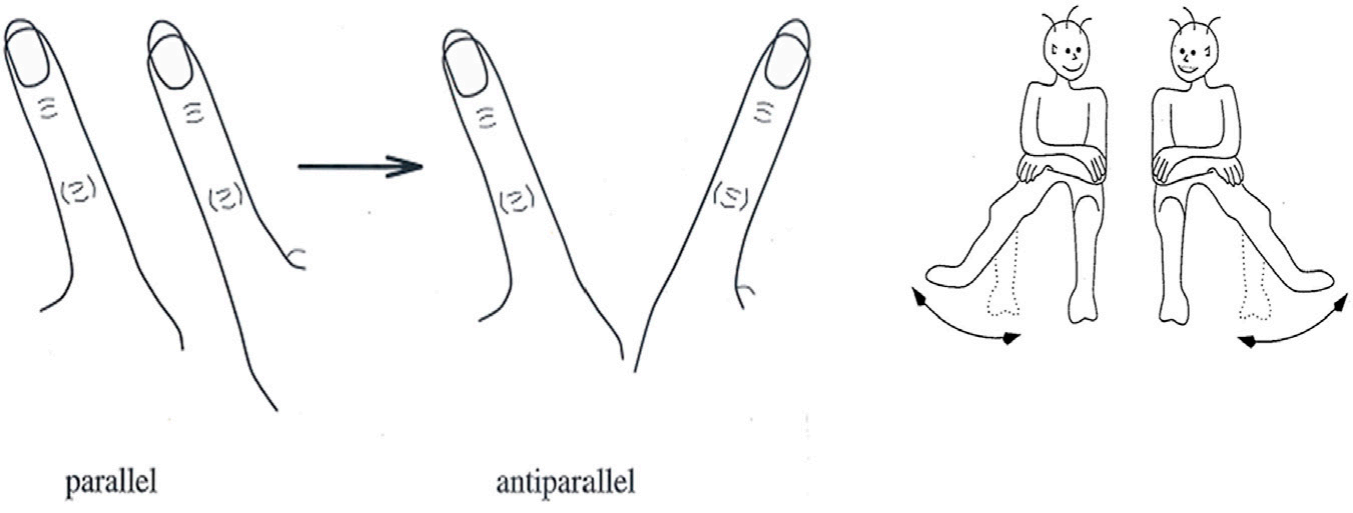
Left: Kelso’s (1984) finger movement experiment. While initially people can move their index fingers in parallel, beyond a critical speed of the finger movements the relative position of the fingers switches involuntarily to the antiparallel, i.e., symmetric, position. Right: The Schmidt et al. (1990) leg movement experiment with results identical to the above Kelso’s experiment.

The theory of synergetics started with what in retrospect Haken termed the first foundation of Synergetics, that is, with the laser paradigm as described above and illustrated in Figure 2 left. Then, Haken applied its basic dynamics to the Bénard (1900) pattern formation in liquid thus forming the synergetics’ pattern formation paradigm (Figure 5 left) and, in order to capture the cognitive process of pattern recognition, he demonstrated that pattern recognition is analogous to pattern formation in the mind-brain (Figure 5 right). This became synergetics’ pattern recognition paradigm. Fourth, the finger movement paradigm was developed (Figure 6) extending the theory of synergetics to the domains of human behavior and coordination dynamics that gave rise to the HKB model (Haken et al., 1985). Common to the four paradigms is that their sources of inspiration were material phenomena as developed and studied in physics and in this respect Synergetics first foundation was similar to all other complexity theory.

But there was one property in Synergetics that from the start differentiated it from the other complexity theories – the top-down process termed by Haken (1988) “the slaving principle”: once emerged, the order parameter enslaves (i.e., determines, describes and prescribes) “the behavior of the individual parts (like a puppeteer who lets the puppets dance)” (Haken, 2016, 151).

This has eventually led Haken to the realization that while cognitive and brain systems are complex, their dynamics is essentially top-down. This is so since as complex self-organizing systems cognition and brain are active systems–a kind of “inference machines” that ongoingly top-down initiate predictions about the environment, comparing them to bottom–up information by means of embodied action-perception (Haken and Portugali, 2021, Chap. 7).

**FIGURE 7:**
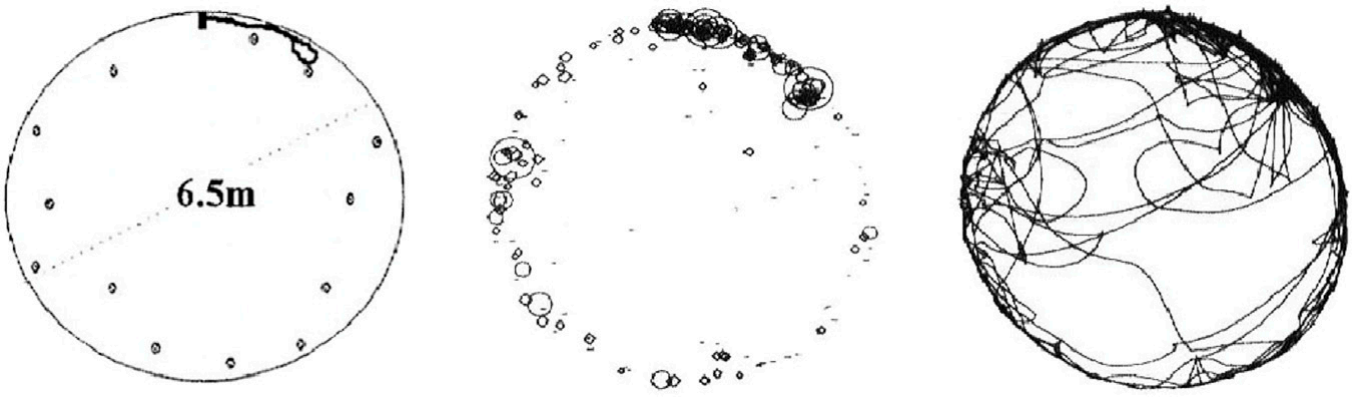
A typical rat’s exploratory behavior experiment (Golani et al., 1997; Golani et al., 1999). *Left*: Line tracing the rat’s forward and backward movements in the first exploratory excursion. Right: Line tracing the rat’s movement in all progressing episodes. Center: The rat’s constructed space (arena): stopping locations (bases) with dwell time represented by circles’ size.

A case in point comes from the domain of ethology regarding the phenomenon of *exploratory behavior*. Studies and experiments in this field show that when an animal (e.g., a rat) is being introduced to a new environment (an empty arena in the experiment below), its first instinctive reaction is to actively start a process of exploration (Golani et al., 1997; Golani et al., 1999). Figure 7 illustrates a typical exploratory behavior experiment with rats; other species perform this process in other ways. And as demonstrated by Munk-Vitelson, (2005) and by a follow-up study (Frostig et al., 2020) humans too perform exploratory behavior when first introduced to a new environment. The common denominator in the various exploratory behavior studies is that this is a phenotypic behavior – an active top-down process, that to my mind leads to niche construction (see below).

From the above follows, firstly, that the first foundation of synergetics refers to systems without memory, whereas its second foundation to systems with memory. Secondly, that when dealing with physical-material systems, the disintegration process is essentially random. This is the way, for instance, a small world network develops, as do the connections and nodes in Barabási’s (2002) and Watts’ (1999) formulations. However, when dealing with systems that possess memory, such as certain living systems, and especially human and social systems characterized by the personal memory of individuals and the collective-historical memory, the situation is different: In these contexts, structures and patterns from the past, constructed as they were in and by the mind-brain, play an important role in both the process of SOD. I’ll further refer to the role of memory in Sect. Five below; however, before doing so it is appropriate to introduce two case studies of SOD: one on the first appearance of cities in the ancient past and the second from a current extreme event.

## 5 Two case studies of SOD

### 5.1 The view from the periphery

The first recognition of the lacuna noted above and thus of the distinction between SOI and SOD followed a study of the first urban revolution in the ancient world and the realization that there are two ways to look at this event: From the core and from the periphery (Portugali, 2023). The perspective on the city and urbanism from the cores of the ancient civilization of Mesopotamia presents a scenario in which about 5,500 years ago, an urban revolution occurred in which cities and urbanism first emerged, and from then until today, the city and urbanism have been integral parts of social reality. Such a view was suggested by Childe’s (1950) seminal paper who first coined the notion of “Urban revolution”.

**FIGURE 8:**
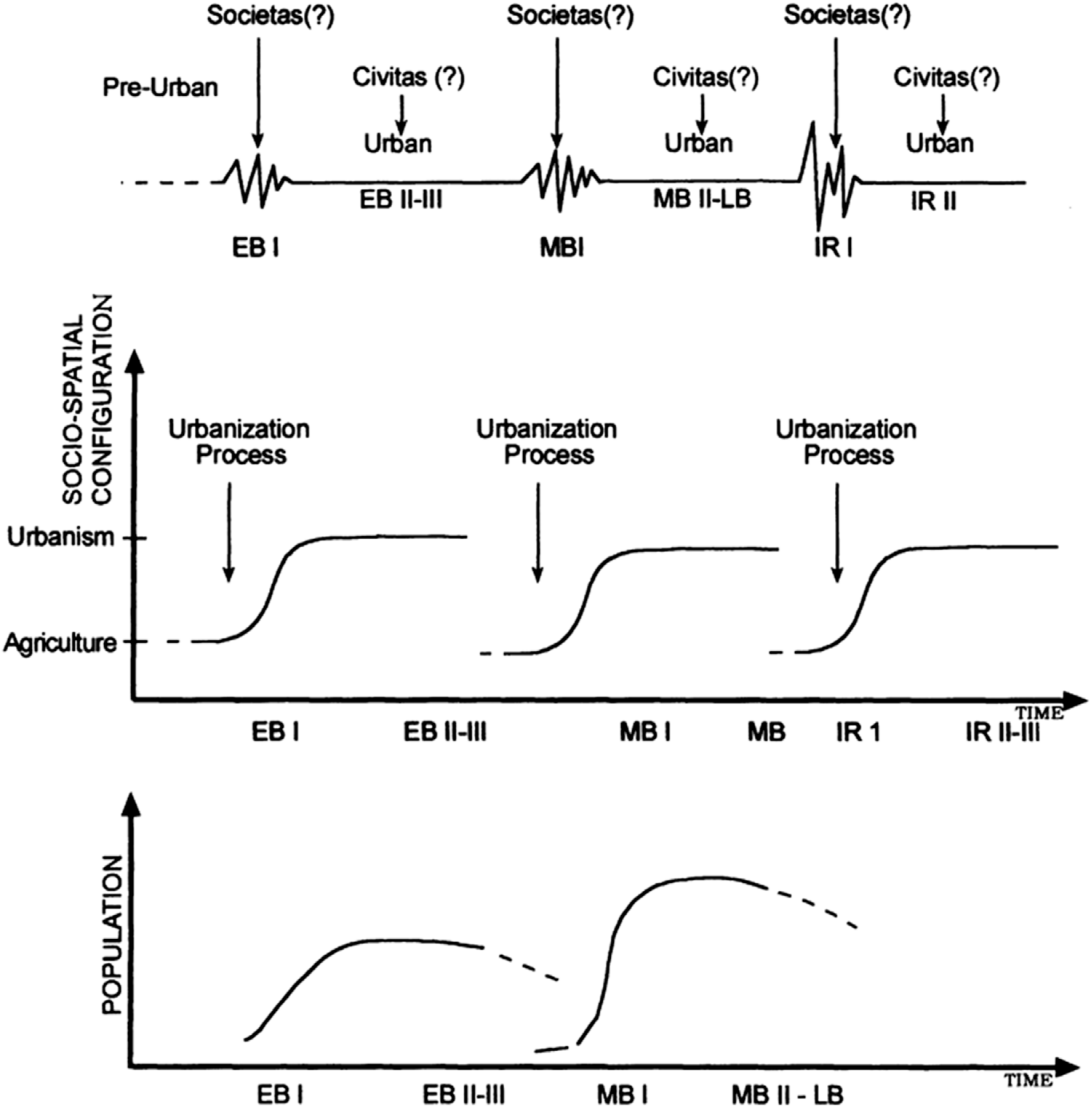
The evolution of the settlement system in the Land of Israel from the Early Bronze Age to the Iron Age. *Top:* A description in terms of a complex system moving between long periods of structural stability, which are interrupted by short periods of chaos and sharp fluctuations. *Middle*: A description of the process as a rhythm between agriculture and urbanism, interrupted by global collapses of the urban system. Bottom: Population changes as calculated for the Early Bronze Age and Middle Bronze Age, according to (Portugali and Gophna, 1993).

The view from the periphery of the land of Israel, as depicted in Figure 8, offers a completely different view (Portugali ibid): three local urban revolutions, three urban cultures, and two systemic collapses—the first following the first local urban revolution and second following the second local urban cultures. The point I want to emphasize is that every urban wave signifies an urban revolution, not in the sense that the city first appeared in human history (as in Mesopotamia), but in the sense that for any non-urban pastoral society, transitioning to an urban socio-spatial structure means a social upheaval in every sense of the word. The current crisis of Bedouin society in Israel, especially those residing in the Negev, is a contemporary example that partially illustrates the traumatic significance of the transition from a pastoral society to an urban one. The fact that this is indeed a revolution, can be appreciated in light of the theories of social evolution (Portugali, 2023, Chap. 2). In each case, it is a transition from a communal society, primitive communism, or an egalitarian society to a political, stratified, and unequal urban society, or similar transitions. Furthermore, the peripheral perspective offers three universal insights into the role of collective knowledge and memory in processes of change in general and specifically in settlement processes.

First, as we have seen, knowledge in a nomadic society about alternative forms of existence (such as urbanism) does not bring about change; change is not a result of choice, but rather of a compulsion. Societies undergo revolutions grudgingly, out of no-choice.

Second, societies that have undergone an urbanization process, in the cases before us, transitioning from a nomadic pastoral life to a sedentary urban one, did not forget their past. The previous forms of life did not disappear but remained “trapped/captive” in their collective memory. As we have seen, when conditions changed, these societies “reverted” to their previous state – a process that we’ve termed the *captivity principle* (see below).

Third, and following on from the previous point, there is a resemblance or symmetry between the process of the formation of an urban society (and perhaps culture in general) and the process of its collapse: in both cases, the society extracts from its knowledge and memory an alternative form of existence, which it had not previously implemented. In the process of the urban revolution, the society extracts the knowledge of an urban society, while in the process of the urban society’s collapse, it extracts the knowledge of its past as a nomadic-pastoral society.

The view from the periphery as depicted in Figure 8 illustrates a cyclical process, in which a large urban wave flows over the land, connecting and integrating its settlements into the global urban network dominated by the centers in Egypt or Mesopotamia, existing in a steady state for a relatively long period and then recedes due to processes of disintegration and nomadization. This cyclical process exposes a tension between two forms of self-organization: self-organized integration versus self-organized disintegration. Once we have identified the possibility that the self-organization process may also lead to, or be associated with, disintegration, we can identify many such processes not only in the ancient world but also in the modern one. For example, the self-organized disintegration of the Soviet Union and Yugoslavia at the end of the 20th century, and of Muammar Gaddafi regime in Libya in 2011. Does this phenomenon of self-organized disintegration can also be found in other areas such as in biological phenomena, cognition, brain dynamics, and more?

### 5.2 Population movement under extreme events

The 2010 earthquake in Haiti – a powerful 7.1 magnitude quake–devastated the capital Port au Prince and claimed 300,000 lives and much more. Following the disaster, researchers Lu et al. (2012), in a PNAS paper, reported on a study that followed the city’s Port au Prince ‘population behavior and movement.

Using mobile phones’ “big data”, immediately following the disaster, the authors were surprised to find out that the widely held view that such a large-scale urban extreme event must cause a systemic chaos of people moving irregularly while fleeing unrest and searching for material support, is wrong. Instead, their quantitative analysis of the data showed the people’s movements were highly ordered, influenced by their historic behavior and social bonds. In Lu’s et al. (2012) words:

“… the results force a change in our conceptualization of disasters as fundamentally chaotic events. People’s movements are highly influenced by their historic behavior and their social bonds, and this fact remained even after one of the most severe disasters in history.”

In a follow-up PNAS paper Kenett and Portugali (2012) further showed, that immediately following the extreme event the first instinct of the city’s population was to return to, or resume, their routinized spatial movement before the event. The explanation to this finding is that

Routinized action, including movement in space, is … a basic property of human behavior; in fact, humans have no choice but to act routinely. [Extreme event] break the routines of daily life and movement in space, but not this basic human need. After an [extreme event], the natural tendency of people is thus to go back to the previous routines and if this is not possible to develop alternative (temporary or permanent) routines. (ibid 111473)

## 6 Memory and systemic collapse

“Memory”, write Zlotnik and Vansintjan (2019), 2, following Squire, (2009) “is today defined …. as the faculty of encoding, storing, and retrieving information”. – A faculty that comes “in multifarious forms and within each form there are multiple dimensions” (Roediger III et al., 2001). As such memory is central to the study of brain, cognitive and behavioral processes in general as well as to synergetics and CTC (Portugali, 2025). As shown by Portugali (2005), different forms of memory imply different forms of cognitive maps and thus different forms of urban agents’ behavior in cities (Portugali, 2016; Haken and Portugali, 2021).

Note that in the above two case studies of SOD, human memory plays a central role–a finding that has led to a realization that theories of complexity and self-organization can be divided into two groups (Portugali, ibid. Chap. 3): theories referring to processes of self-organization without memory and theories referring to processes of self-organization with memory. Examples of self-organization without memory include processes like the laser paradigm in Haken’s theory of Synergetics (Figure 2 left) the heat convection process in Bénard cells (Figure 5 left), and Prigogine’s dissipative structures theory (see Portugali, 2011 for Introduction and examples). In contrast, an example of self-organization with memory is the paradigm of pattern recognition in Haken’s theory of synergetics (ibid).

Several characteristics are specifically important in a self-organization process with memory. Firstly, information from the past and the existing knowledge in memory are part of the players or forces involved in the process. Secondly, in a system with memory, there are instincts, tendencies, or default ways to deal with any new phenomenon, situation, or problem, primarily through patterns or solutions stored in memory. Only when these defaults fail, does the system start generating new solutions. A clear example of this is the process of vision or the pattern recognition process.

Thirdly, as described here and expanded in Haken and Portugali (2021), according to the theory of Synergetics, a new structure emerges out of the interaction between parts of the system, leading to the creation of an order parameter that once emerges enslaves other competing order parameters by canceling them. That is, in a system without memory, competing parameters or past control parameters are simply erased. In a system with memory, the process occurs through the *imprisonment principle* or *captivity principle* (Portugali, 2023, Chap. 13): Following the enslavement process, alternative configurations and solutions are not lost but are kept in memory in a state of “imprisonment”/“captivity”. This means that when the “prison walls” weaken or are breached, these configurations are released and start to act.

## 7 Epilogue: the unfinished study

As noted at the Prolegomenon to this paper, our discussions (of Haken’s and myself) regarding SOI vs. SOD was never completed and ended more or less at this point. However, in parallel we’ve developed several other topics, in particular the notions of *synergetic inter-representation networks* (SIRN), *information adaptation* (IA) and their conjunction SIRNIA and the view that cities are hybrid complex systems (HCS). Had we had the time to further elaborate the SOI vs SOD issue, it would probably be by relating it to the latter notions. In what follows, I concisely introduce these notions and conclude with preliminary implications regarding the way they are related to the process of SOD.

### 7.1 HCS–Hybrid complex systems

The domain of complexity theory started by reference to material complex systems such as the phenomena of Laser and Bénard cells. Accordingly, CTC too started by demonstrating that cities are analogical to such material complex systems. At a later stage, following the introduction of complex adaptive complex systems (CAS) by Gell-Mann (1994) and Holland (1995), the domain of CTC responded by showing that cities are CAS too. However, as shown in several studies (Portugali, 2016; Haken and Portugali, 2021) cities still qualitatively differ from CASs in that cities are hybrid complex in several respects: First, in that they are composed of artifacts^1^ which are simple systems (buildings, roads) and human agents, each of which is a complex system; and, it is due to the human urban agents that the city is complex (Portugali, 2016). Second, in that as CAS the urban agents adapt not only by means of their behavior but also by the production of artifacts. Three, artifacts are the staff of cultural evolution and as a consequence, cities are subject to two processes of evolution: the very slow Darwinian evolution (like all animals) and the extremely fast cultural evolution (Portugali, 2011). Forth, and as a consequence, while DNA is central to organic evolution, human memory is at the core of cultural evolution. *SOD in this respect is a property of HCSs*.

The view of cities as HCS evolved gradually out of the attempt to study the behavior of urban agents and thus of the dynamics of cities from the perspective of cognition and brain dynamics. It turned out that while this attempt entailed important insights (e.g., agents behave according to cognitive maps) it also exposed that the cognitive science fails to acknowledge the role of artifacts which as just noted, in the case of cities, are central to the dynamics. This situation has led to the development of a sequence of notions that consider artifacts as integral elements in the cognitive process, namely, to synergetic inter-representation networks (SIRN), information adaptation (IA) and their conjunction SIRNIA.

### 7.2 SIRNIA—A conjunction between SIRN and IA

The notion and model of SIRNIA evolved gradually, as noted, out of a sequence of studies referring specifically to urban dynamics of cities as HCS. The motivation from the start was to devise a model of an urban agent which, as implied by HCS, adapt not only by means of behavior, but also by the production of artifacts. First, the *inter-representation nets* (IRN) was suggested (Portugali, 1996) and reformulated in terms of the synergetic computer as SIRN (Haken and Portugali, 1996). Next, it was shown (Haken and Portugali, 2003) that “the face of the city” affects urban agents’ behavior and action by means of three forms of information it conveys: quantitative Shannonian information (SHI) and two forms of qualitative information–semantic information (SI) and pragmatic information (PI). Third, the process of *information adaptation* (IA) was developed (Haken and Portugali, 2015) and finally their conjunction in the SIRNIA model (Haken and Portugali, 2021).

**FIGURE 9:**
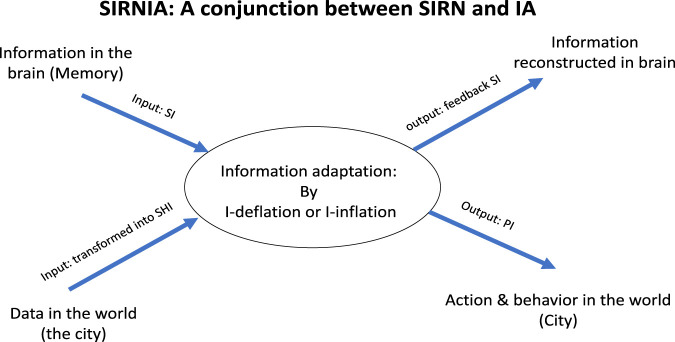
The SIRNIA model. For details see text.

The SIRNIA model illustrated in Figure 9 symbolizes a complex self-organizing urban agent that is ongoingly subject to two flows: external and internal. The first is a flow of data coming from the ‘world’ (the city in the present context) – *via* the senses, the agent’s body and/or artifacts; whereas the second is a flow of information coming from the agent’s mind/brain, in the form of ideas, fantasies, dreams, thoughts, memories and the like. The interaction between these two flows gives rise to an order parameter that by means of pragmatic information (PI) governs the agent’s action and behavior, as well as the feedback semantic information flow (SI) it restructures the agent’s mind-brain.

**FIGURE 10:**
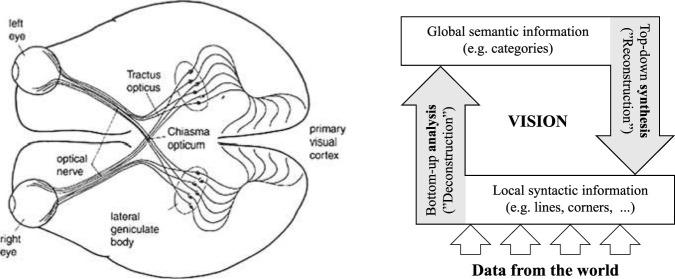
The process of vision: *Left*, the links between the eyes and the brain; *right*, a schematic description. The process evolves as follows: data from the world is first analyzed by the brain, in a bottom-up manner; this local information interacts with a top-down process of synthesis that gives rise to global information—that is, to seeing and recognition (See Haken and Portugali, 2015).

**FIGURE 11:**
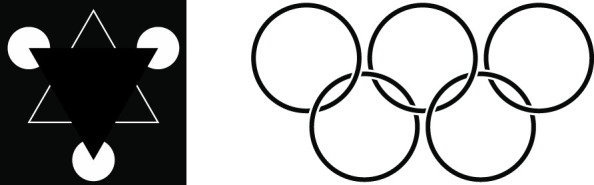
Left: The ‘Kaniza triangle’ illusion serves as an example of information adaptation implemented by means of information inflation, in which we see lines where there are none; our brain adds virtual line where no lines exist to mark out intersecting triangles. *Right:* The “Olympic rings” illusion serves as an example of information adaptation implemented by means of *information deflation*: we see five circles in superposition and overlook the many geometric forms of which this figure is also be composed.

The order parameter emerges by means of a process we’ve have termed (Haken and Portugali, 2015) *Information adaptation* (IA) illustrated in Figure 10, with respect to vision: data from the world analyzed by the mind-brain in a bottom-up manner interacts with a top-down flow of information constructed in the mind-brain-body, giving rise to seeing and recognition. For this to happened the two flows should be adapted to each other and this process of information adaptation is being implemented by means of *information inflation* (as in Figure 11, *left*), and/or *information deflation* (as in Figure 11, *right*). (For more details and further references see Haken and Portugali, 2021).

### 7.3 Preliminary implications

SOI is a property of complex material systems (i.e., without memory) on the basis of which the synergetic first foundation was developed, while SOD is a property of complex living systems (with memory) on the basic of which the second foundation of synergetic was developed. However, as implied from the above, complex living systems can further be divided into natural/biological living vs hybrid systems/HCS: The first are subject to the slow Darwinian evolution while the second to both: the slow Darwinian evolution and the extremely fast cultural evolution. SOD is clearly a property of the relatively fast cultural evolution (can it be found also in natural evolution?). to the latter the SIRNIA model further adds that SOI and SOD are two forms of IA: when the control parameter is in a growth mode, the dynamics leads to SOI; when in a decay mode, to SOD.

The notion of HCS is thus built, firstly, on the interface between complexity theory and cultural evolution – an issue that had attracted some preliminary (Baskin, 2013), yet (to my mind) not sufficient discussion, that has yet to be extended. Secondly, on a distinction between cultural and natural/biological evolution – a controversial issue, the literature on which is huge and there is no room to refer to it here.

As one of the reviewers of this paper commented, “[t]his unfinished opening underscores the need for an integrative theory of urban complexity that combines synergetics, distributed cognition, and network theory, offering new conceptual tools for understanding the emergence and transformation of urban systems.”

## 8 Concluding notes

On 27 March 2025 a webinar entitled “network physiology in extreme events” took place^2^, focusing on

“how humans adapt to and survive in environments [that] include conditions that deviate significantly from what is considered normal or optimal for life, such as extreme temperatures, extreme pressures, or limited oxygen availability … Understanding the integrated physiological response to these extreme conditions is essential for optimizing performance, … and advancing our knowledge of human adaptability and resilience under stress … “.

What is missing in this statement is humans’ main means of adaptation—the production of artifacts that enable humans to ….“adapt to and survive in environments [that] include conditions that deviate significantly from what is considered normal or optimal for life, such as extreme temperatures, extreme pressures, or limited oxygen availability … ” and, unlike the rest of animals, all these without significant transformations in their network physiology.

Furthermore, as noted above with respect to synergetic cities, as complex self-organizing systems cognition and brain are active systems–a kind of “inference machines” that ongoingly top-down initiate predictions about the environment, comparing them to bottom–up information by means of embodied action-perception (Haken and Portugali, 2021, 88, 91). Active inference is central also to network physiology as implied by a recent study (Friston et al., 2025) and in another study Pezzulu et al. (2024) write that “Active inference is based upon the idea that sentient behavior depends upon our brains’ implicit use of internal models to predict, infer, and direct action.” A similar process takes place in the two case studies of SOD described in Sect. Four above: In the case of ancient urbanism, as the control parameter (the control of the Egyptian center) decreases and becomes weaker, internal models stored in collective memory were extracted and applied to reality. Similarly, in the case of the extreme event, the internal model of daily spatial routine was used “to predict, infer, and direct action.”

As we’ve seen above, the process of SOD is a property of hybrid complex systems. And as noted by Simon (1969) in the preface to the second edition of his *The Sciences of the Artificial*, “the contingency of artificial phenomena has always created doubts as to whether they fall properly within the compass of science”. As a consequence it is not surprising that the common practice is to study the human organism independent of its artifacts production capabilities. But is this justified?

Two decades ago the notion of *niche construction theory* (NCT) was introduced (Odling-Smee et al., 2003; Laland et al., 2016; Constant et al., 2018) suggesting that the constructed niche environment not only protects the animal, but also feeds back and thus participates as an integrated component in the organism’s evolutionary processes, e.g., in its process of natural selection–a view that entailed much debate in biology. Can NCT be extended to the domain of cities? In a recent study (Portugali, 2025) it was suggested that, as noted above, one of the key difference between animals’ natural niches and humans’ artificial environments is “the fact that natural niches are subject to the slow process of Darwinian evolution, while artifacts are the stuff of cultural evolution and are, thus, subject to the fast process of cultural evolution.“. This issue entails several other ones: What is the physiological network responsible for the production of artifacts? Do artifacts affect the human physiology and if they do -how? Should the domain of NP include artifacts’ production? Interesting as they are these and similar questions will have to await further research.

## Data Availability

Publicly available datasets were analyzed in this study. This data can be found here: Not applicable.

## Footnotes

1 Rooted in Aristotle’s distinction between things that exist by nature and things that exist by craft, the notion “artifact” refers to “objects made intentionally, in order to accomplish some purpose” (Stanford .).

2
https://www.physoc.org/events/network-physiology-in-extreme-environments/
